# Therapeutic and diagnostic implications of exosomes as natural nanoparticles: a new paradigm in brain cancer disease management

**DOI:** 10.3389/fmed.2025.1599392

**Published:** 2025-07-21

**Authors:** Shanid Mohiyuddin, Pankaj Dipankar, Belfin Robinson, Andrew Jeyabose, J. Karthikeyan

**Affiliations:** ^1^Department of Medicine, University of Missouri, Columbia, MO, United States; ^2^National Institute of Nursing Research, National Institutes of Health, Bethesda, MD, United States; ^3^Department of Neurology, School of Medicine, University of North Carolina, Chapel Hill, NC, United States; ^4^Department of Computer Science and Engineering, Manipal Institute of Technology, Manipal Academy of Higher Education, Manipal, India; ^5^Department of Software and Systems Engineering, School of Computer Science Engineering and Information Systems (SCORE), Vellore Institute of Technology, Vellore, India

**Keywords:** exosome, brain cancer, theranostic, iRGD-peptide, Blood-Brain Barrier, extracellular vesicles, nanoparticles, liquid biopsy

## Abstract

The clinical translation of safe and effective therapeutic methods for brain cancer treatment is a major challenge that persists in modern medicine. The insufficient drug delivery into the regime of the affected brain tissue due to blood–brain barrier (BBB) restriction leads to a poor prognosis of the disease. However, an alternative strategy using biomaterials like exosomes can offer advancements in the treatment of brain cancer. Exosomes are a type of extracellular microvesicle with a diameter of 30–100 nm, principally functioning as intercellular mediators in the cell signaling process. Due to their biological origin, exosomes demonstrate a significant ability to cross the BBB and possess enhanced biocompatibility, high drug-loading capacity, and low immunogenicity. With the innate property of biomolecule delivery, exosomes also offer enhanced cellular uptake, rendering them exceptional in drug delivery systems. Herein, we focus on the anticancer and diagnostic applications of exosomes for brain cancer therapeutics. The enhancement of the physico-chemical properties of various cell-derived exosomes can be effectively used as a prime drug delivery agent in most treatment strategies. The biphasic and fast drug release in acidic pH of the tumor microenvironment by exosome-mediated drug delivery system contributes to passive targeting, which is often considered advantageous over other drug delivery platforms. These characteristic features are likely to enhance the therapeutic potential and efficacy of the treatment. The exosome loaded with the drug acts as an efficient biomaterial to surpass the BBB, followed by efficient cellular uptake, leading to cytotoxicity in glioblastoma cells. In this review, we summarize the recent updates in theranostic and prognostic strategies using exosomes as a mediator and their prevalence in biomedical applications, with a focus on brain cancer diseases.

## 1 Introduction

Exosomes are nanoscale extracellular vesicles, typically 40–100 nm in diameter, distinguished by their well-defined physicochemical properties. They play a pivotal role in intercellular communication through the transfer of bioactive molecules and modulating key cell signaling pathways (1). The bio-generated exosomes secreted by cells after fusing with multivesicular bodies are released into the extracellular matrix. They further transcend to neighboring or distinct cells, promote the delivery of information in the form of proteins, lipids, and Ribonucleic acid (RNA), which involves *de novo* transcriptional and translational changes in the host cells (2). Exosomes are readily found in most extracellular fluids, both in physiological and pathological conditions (3). The isolation of exosomes involves classical differential ultracentrifugation and density gradient ultracentrifugation (4, 5), size-based isolation or ultrafiltration (6), microfluidics-based isolation (7), exosome precipitation (8), and immune-affinity-based capture (9) from the body fluids. The intrinsic functional property of exosomes in transferring material through the cells evidently increases cellular uptake (10) in both *in vitro* and *in vivo* systems. Moreover, the enhanced biocompatibility of biologically derived exosomes (11), as well as the better transfer of cargo to the cell, and the abundance of body fluids, relatively increase the implementation of exosomes in the drug delivery system. The ease of isolation and biological source of origins are also contributing much to the successful employment and providing a better platform in the drug delivery system.

The blood–brain barrier (BBB) is a distinguishable lining boundary of cells in the vasculature of brain tissue, functioning as a highly selective barrier that separates blood and brain fluid. BBB primarily regulates and directs the nutrients and other essential biomolecules (12) to the brain. For a successful treatment of brain tissue-related diseases, the therapeutics should reach the regime of the brain tissue. However, the strict nature of the BBB restricts therapeutics from crossing the barrier, resulting in a poor prognosis of the disease. The bioavailability of biomolecules, drugs, small molecules, and other metabolites in the brain is often poor due to functional aspects of the BBB (13, 14). To address this issue, the practice of ultrasound and other strategies temporarily opens the BBB, which yields a surpass of the drugs/payloads across the barrier, leaving structural impairment to the BBB (15). Traditional drug delivery system has certain disadvantages, including low accumulation and bioavailability in target cells, off-target toxicity, and quick elimination, which restricts their therapeutic interventions (16). Thus, a controllable and non-harmful way of transporting the cargos across the BBB is the need of the hour. Nanoparticle-based delivery approaches have been found to be ideal in this scheme with enhanced efficiency and potent targeting to the specified location.

Over the past few decades, numerous synthetic delivery vectors, including liposomes, polymer micelles, inorganic nanoparticles, and dendrimers, have been synthesized to address these difficulties in clinical translation. Liposomes are one of the effective and clinically authorized vectors in the nanoparticle domain currently available (17). Liposomes are bi-layered membranes made of lipids, which are effective in loading hydrophilic drugs into the liposome lumen. When engineered, targeting ligands can be added to improve tissue uptake. Although liposomes are lipid-soluble, due to the size and the presence of tight junctions between BBB cells, liposomes are restricted from easily diffusing across the BBB (18). To get across the BBB, they must instead use certain transport mechanisms or techniques like ultrasound-mediated delivery (19). Polymeric nanoparticles, inorganic nanoparticles, and dendrimers also confront several obstacles, such as the lack of organ-specific targeting, physical and chemical traits associated with toxicity, and an adverse immune response (20), also contributing to deficiencies in the imperative cellular uptake and BBB crossing to treat brain cancer.

Exosomes, also known as natural nanoparticles, are best suited for drug delivery applications in brain-related diseases. The first discovery of exosomes dates back over 25 years, when researchers found the formation of extracellular vesicles, involved in the selective externalization of the transferrin receptor (16). However, exosomes can acquire a higher degree of complexity in individual components; they are rich in phospholipids, cholesterol, and sphingomyelin. Furthermore, extra binding sites for drug loading may occur in exosomes due to biomolecules present in the membrane and core (20). In recent years, there has been a multi-fold increment in the successful implementation of exosomes as a drug delivery vehicle in biomedical research. The increasing attention in exosome research highlights its unique physico-chemical properties as a natural nanoparticle and has revolutionized the diagnostic and therapeutic regime in brain cancer patients. Yang et al. reported a drug delivery system with exosomes for doxorubicin (Dox) across the BBB in zebrafish (*Danio rerio*) model, with improved cellular uptake of the drug (21). The exosomes encapsulate therapeutic payloads, such as chemotherapeutic drugs and biomolecules, warranting the effective delivery to the brain tissues, enabling local therapeutic effects while reducing non-specific and systemic side effects (18, 19). The biological origin and composition of exosomes improve the immune tolerance and increase biocompatibility compared to synthetic nanocarriers (22). Furthermore, exosomes can be considered a prime contender in the diagnosis of diseases through various biomarkers in brain cancer conditions, by non-invasive methods (from body fluids such as sweat, urine, and saliva) or other modes like liquid biopsies. Moreover, the application of surface-engineered exosomes as therapeutic carriers has emerged as a promising strategy to unravel the limitations of conventional drug delivery systems. By exploiting the intrinsic cellular uptake and circulation mechanisms of exosome-derived pathways, this approach enhances the multitude of targeted drug delivery and therapeutic applications (23).

## 2 Materials and methods

In this review, we conducted a comprehensive literature review and gathered relevant studies for the evidence synthesis. A detailed methodology, which was utilized for the search strategy and inclusion/exclusion criteria, is elaborated in the following sub-sections.

### 2.1 Study design

This review focuses on the current updates in the therapeutic and diagnostic implications of exosomes in brain cancer research and clinical manifestations. This review aims to provide a concise yet comprehensive literature review of the latest advancements and emerging insights into the role of exosomes in the diagnosis, prognosis, and therapeutic interventions for brain cancers, with a special emphasis on glioblastoma and glioma.

### 2.2 Sources database and search strategy

A search for a comprehensive literature survey was conducted in electronic databases, including PubMed, Web of Science (Core Collection), and Scopus. The detailed search strategies for each database are described in Supplementary Table S1.

### 2.3 Inclusion and exclusion criteria

To establish a comprehensive literature review, we performed a search strategy using the keywords and related Medical Subject Headings (MeSH) terms for “brain cancer,” “glioblastoma,” and “exosomes.” We restricted the search to original articles and review articles on exosomes and brain cancer. This search was restricted to peer-reviewed articles published in only English-language journals between 2009 and 2024. The editorials, case reports, conference abstracts, dissertations, gray literature, and non-English language articles were excluded from evidence synthesis.

## 3 Exosomes in brain cancer disease progression

**Figure 1:**
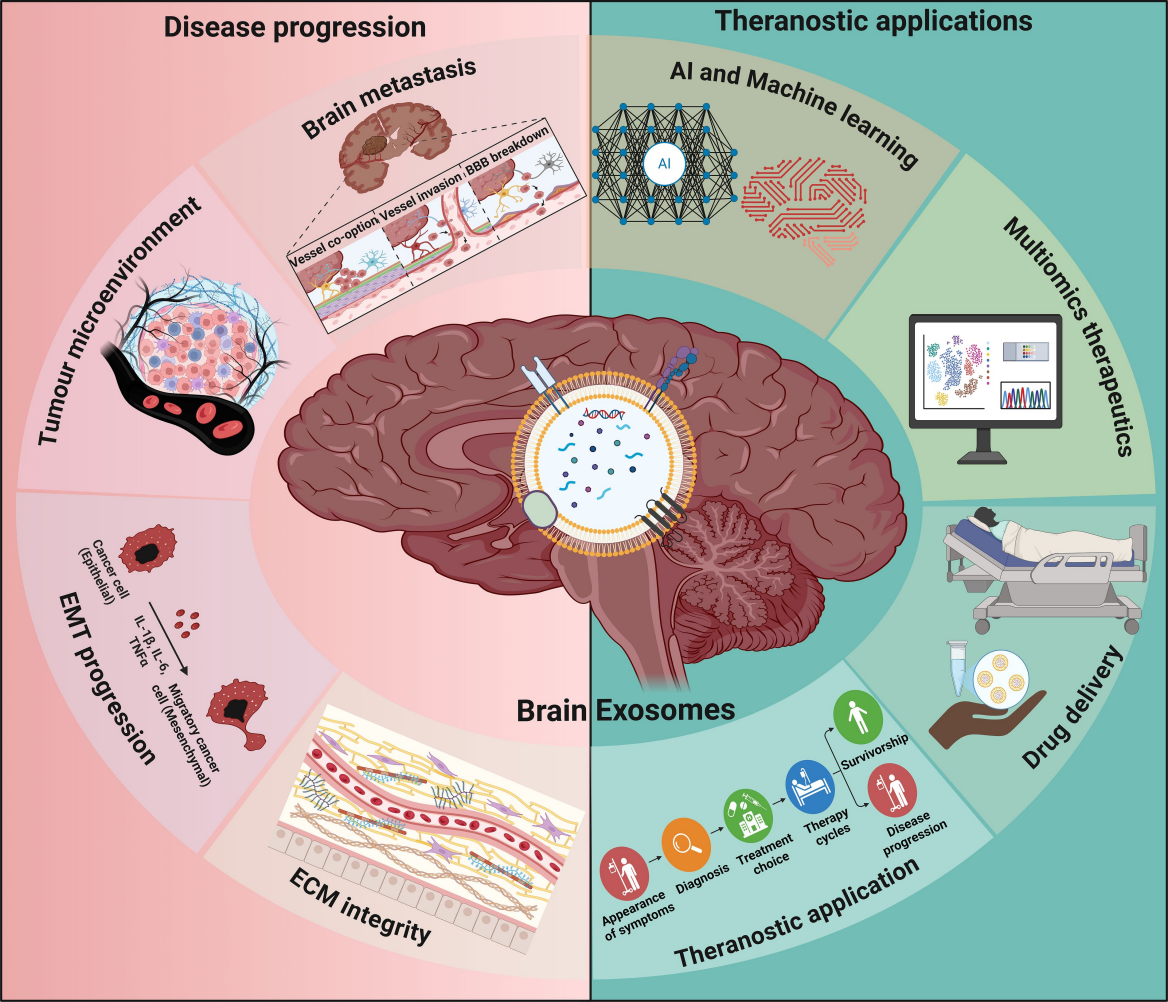
Graphical abstract as an overall conceptualization and review of the literature. Multimodal theranostic implications of exosomes for brain cancer. Exosomes derived from brain tissues can be suitable contenders in applications such as drug delivery, prognostic and diagnostic platforms, and disease progression in the brain cancer regime. The intriguing applications of exosomes range from disease progression markers, such as brain metastasis, tumor microenvironment, epithelial mesenchymal transition, and extracellular matrix (ECM) integrity. However, in therapeutic aspect components, such as machine learning, AI, multi-omics, drug delivery, and other theranostic applications, were covered. The schematic diagram was created using BioRender (https://biorender.com).

Exosomes impart in the regulatory mechanism of pathological angiogenesis, including tumor angiogenesis by actively delivering pro-angiogenic biomolecules like matrix metalloproteinases (MMPs), vascular endothelial growth factor (VEGF), and other microRNAs (24). In a tumor microenvironment, the intra-tumoral hypoxia evidently stimulates angiogenesis and led to improved uptake of tumor derived exosomes. Hypoxia-induced cell signaling in cancer cells is mediated by exosomes (25) and affects tumor growth and metastasis. Due to the protective and selective nature of the BBB, brain metastasis presents a unique challenge in prognosis. However, tumor-derived exosomes are intermediate between the cancer microenvironment and that of healthy tissues in the brain. This further enhances the aggressiveness of the disease, thereby halting the remission. It was observed that tumor-derived exosomes can alter the BBB, promoting an environment favorable for developing secondary tumors at the site (26). Moreover, exosomes carrying specific miRNAs, which regulate cellular responses and modulate tumor-stroma interactions, lead to the induction of metastasis (27). Cancer cells use exosomes as tools to prepare distant sites for metastasis, a phenomenon often referred to as “premetastatic niche” formation. These exosomes can deliver growth factors and other molecules that remodel the microenvironment, making it favorable for metastasis (28). Tumor-derived exosomes usually contain a cell migration-inducing and hyaluronan-binding protein (CEMIP) that can induce pro-inflammatory changes in microglia (M1), later leading to tumor progression in the brain microenvironment (29). Exosomes play crucial roles in transforming the tumor microenvironment, influencing processes such as angiogenesis, immune evasion, and metastasis. In a recent study, exosomes released by tumor cells regulate immune response and disease progression by promoting the M2 macrophage population in the tumor microenvironment (30). Exosomes can mediate the pro-tumorigenic microenvironment by delivering oncogenic signals and promoting the recruitment and activation of various stromal cells (31). Numerous studies further illustrate the capability of exosomes to modify the tumor microenvironment, thereby facilitating metastatic progression. Brain metastatic exosomes disrupt the endothelial tight junction proteins, leading to the compromise of the integrity of the BBB (32, 33). This results in increased permeability, allowing for easier dissemination of metastatic cancer cells. Although exosomes play a profound role in disease development and progression, we can exploit the possibilities of exosomes in therapeutic aspects as well. Exosomes also possess distinct physico-chemical characteristics, as enhanced cellular uptake has a significant impact on theranostic applications. The various implications of exosomes in the disease progression and therapeutic aspects are illustrated in Figure 1 (created using biorender). The schematic illustration describes the multimodal theranostic inference of exosomes in brain cancer disease. Exosomes derived from brain tissues can be used as a leading contender in applications platform, such as drug delivery, prognostic and diagnostic platforms, and disease progression marker identification.

## 4 Theranostic implication of exosome in brain cancer

Glioma is primarily considered a brain tumor. With respect to the cell type of origin, they are sub-classified into ependymomas, astrocytomas, anaplastic astrocytomas, glioblastoma, oligodendrogliomas, and mixed gliomas (34). Among them, Glioblastoma multiforme (GBM) is considered the most aggressive and frequent (60% of total brain tumors) form of malignant tumor in adults (35). Irrespective of advancements in modern therapies, GBM continues to be a deadly disease with a high mortality rate, poor prognosis, and low survival period in the medical field. Although the incidence rate of GBM is comparatively less with respect to other cancer types, GBM stands as the third foremost cause of cancer-related mortality in the age group of 15–34 years (36). The combined insights from studies on exosome-derived mechanisms and their advancements in engineering introduce innovative approaches in glioblastoma management. The therapeutic applications range from targeted delivery to immune modulation, reflecting the dual role of exosomes as both carriers and modulators of treatment strategies (37). Consequently, integrating exosome-based methodologies will open a new paradigm in precision oncology and brain cancer therapy.

**Figure 2:**
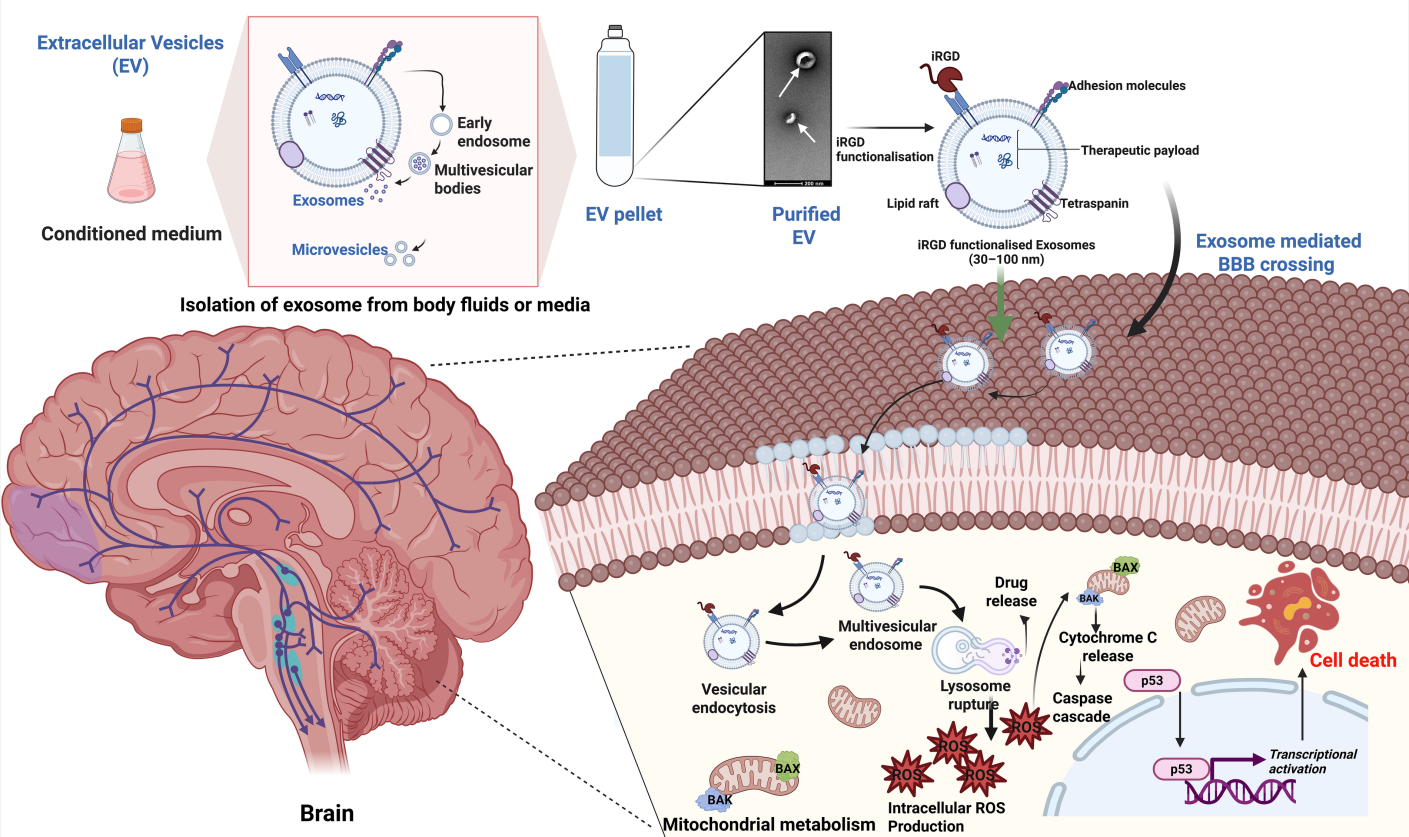
Multimodal targeting of iRGD-mediated active targeting of brain cancer cells by exosomes. Exosomes isolated from body fluids or cell culture media functionalized with the iRGD peptide as the targeting moiety exhibit enhanced BBB crossing and therapeutic payload delivery in brain cancer cells. Such surface-tailored exosomes release therapeutic payload after vesicular-mediated endocytosis and late lysosomes, leading to burst drug release and mechanistic cell death. The delivery of a therapeutic payload by exosomes improves the activation and magnitude of caspase-mediated apoptotic cell death, and reactive oxygen species accumulation in targeted brain cancer cells can be achieved by iRGD-conjugated exosomes. Also, such therapeutics reduce the non-specific off-target effects and preserve healthy brain cells, and by surpassing the BBB, increase the uptake in the brain tissue. The schematic diagram was created by using BioRender (https://biorender.com).

Conventional chemotherapy is not adequate to compete GBM due to the complexity and heterogeneity in the pathogenesis (38) of the disease. However, surface-engineered exosomes have been found to be efficient drug delivery cargo due to the presence of an active targeting moiety. Modifications using tumor-penetrating peptides, such as the integrin-specific (iRGD) peptide, enhance the targeting accuracy of exosomes to brain tumors (39). The iRGD (CRGDKGPDC) peptide has been an important targeting moiety in recent years for most solid cancers. The iRGD peptide binds to αv integrins and neuropilin-1 (NRP-1), which is overexpressed in glioblastoma and other brain cancers, significantly enhancing the cellular uptake of therapeutic agents (40). HEK-293T cell-derived exosomes, tailored with iRGD, increase the accumulation of therapeutic agents at tumor sites (41). Such a method not only improves drug delivery efficiency but also helps overcome the challenges of the BBB, which typically hinders the accessibility of conventional drugs (39). An approachable multimodal targeting of iRGD-mediated active targeting of brain cancer cells and the delivery of therapeutic payloads can be achieved by exosomes (Figure 2), which improves the activation and magnitude of caspase-mediated apoptotic cell death and reactive oxygen species accumulation in the targeted brain cancer cells. Moreover, such therapeutics reduce the non-specific off-target effects and preserve healthy brain cells, and by surpassing the BBB, they increase the uptake in the brain tissue.

The combination of drug delivery and diagnostic capabilities in engineered exosomes embodies the concept of theranostics, paving the way for tailored treatment plans based on individual tumor profiles. Understanding these mechanisms is crucial for developing therapeutic strategies that target the intercellular signaling pathways modified by exosomes.

## 5 Active targeting approach through iRGD-functionalized exosomes

The surface engineering of exosomes with the iRGD peptide provides an active targeted delivery approach by facilitating specific interactions with the overexpressed αv integrins on tumor cell surfaces, thus improving cellular uptake and penetration into the tumors (42). In recent studies, iRGD-functionalized exosomes have been evidenced as a better delivery probe for chemotherapeutics and RNA therapeutics. The specificity of the iRGD peptide to targets αvβ3/ανβ5 integrins and neuropilin receptors, which are frequently expressed in tumor cells, is essential for entering tumor tissues.

iRGD peptide-functionalized exosomes isolated from immature dendritic cells of mice were loaded with Dox and showed an effective therapeutic index in both *in vitro* and *in vivo* models (39). Macrophage-generated exosomes are used to load paclitaxel drug and show increased toxicity in drug-resistant MDCKMDR1 (Pgp +) cells (43). Exosomes encapsulated with curcumin, along with a STAT3 inhibitor, resulted in a significant reduction in tumor volume in the mouse tumor model (44). In a combinatorial treatment strategy, multifunctional exosomes functionalized with iRGD loaded with Dox and radioiodine-131(131I) can enhance the efficacy of treatments by facilitating deeper tissue penetration and sustained drug release (41). In a current study, a bifunctional iRGD-functionalized exosome loaded with Dox crosses the BBB and elicits a targeted delivery in a therapeutic intervention of central nervous system lymphoma (39).

Despite the promising capabilities of iRGD-functionalized exosomes as a theranostic delivery system, many challenges remain due to their practical clinical implications. The heterogeneous contents among exosomes, probable immunogenicity, and the lack of standardized production methods limit the iRGD-functionalized exosome from clinical administration (45). Further research is required to improve the capabilities of exosome engineering techniques and optimize the cargo for specific brain cancer types. Combinatorial approaches with existing treatment modalities could also enhance the therapeutic efficacy of these exosome-based systems.

Exosomes hold considerable promise in the treatment of brain cancer (illustrated in Tables 1, 2), and several obstacles must be overcome before their clinical implications can be fully realized. These include the clinical translation, safety and regulatory concerns, manufacturing, and scalability. The lack of extensive, randomized controlled trials (RCTs) is a significant limiting factor for exosome therapeutics in clinical practice. Current clinical studies for diseases, including lung and prostate cancer, are still in their early phases, and more research is required to confirm the applicability, safety, and effectiveness of the treatment. Finding the optimal dose, delivery method, and biomarkers to forecast the response of exosome therapeutics is a key hurdle. Furthermore, the lack of a comprehensive understanding of the long-term effectiveness and off-target effects, which is important for developing uniform treatment procedures (46) Moreover, careful planning of clinical trials to assess both the possible hazards of long-term usage as well as the immediate therapeutic advantages will be necessary to overcome these obstacles (47).

**Table 1:** Overall summary of multimodal theranostic implications of exosomes for brain cancer.

Theranostic aspects	Role/component of exosome	Mechanism of action	Implications of the study	References
Diagnostic marker	miR-210, miR-301a	Elevated in serum exosomes; correlates with glioma grade and chemoresistance	Non-invasive grading, prognosis, and prediction	Hamzah et al. (78), Naryzhny et al. (79), Azambuja et al. (80)
Drug delivery	Engineered exosomes with chemotherapeutics	Cross BBB and surface modifications (angiopep-2)	Targeted delivery of TMZ, siRNAs, or DNA repair inhibitors in cancer	Yu et al. (59)
Drug resistance overcoming	miR-21/TLR8-NF-κB pathway inhibition	Downregulates PTEN and TERF1; blocks exosome-mediated resistance signals	Reverses TMZ resistance in glioma	Yasamineh et al. (81)
Immuno-modulation	TNC protein, miR-1246	T-cell suppression by α5β1/αvβ6 integrins, M2 macrophages polarization	Immunosuppressive microenvironment, immunotherapeutic targets	Bamodu et al. (82)
Metastasis monitoring	Exosomal miR-375	Correlates with brain metastasis in neuroblastoma	Biomarker for metastatic progression and monitoring	Wu et al. (83)
Radiotherapy enhancement	Catalase-loaded M1Exosome + anti-PD-L1	Alleviates hypoxia, inhibits DNA repair, and blocks PD-L1-mediated T-cell suppression	Synergizes with radiotherapy to improve glioma outcomes	Rodrigues et al. (84)
Tumor microenvironment remodeling	Astrocyte-derived miR-19a	Transfers miR-19a to tumor cells, suppressing PTEN and myeloid cell recruitment	Promotes metastatic outgrowth; target for microenvironment normalization	Valadi et al. (85)

Brain tissue-derived exosomes have applications in drug delivery, prognostic and diagnostic platforms, disease progression, and biomarker or disease monitoring platforms. This table summarizes the theranostic aspects, roles, or components of exosomes; mechanisms of action of exosomes; and implications of this study.

**Table 2:** List of reported exosome-mediated applications in theranostic platforms for brain cancer.

Main focus	Key findings	Implications in theranostics	References
Exosomes and blood–brain barrier integrity	Exosomes from brain metastatic breast cancer cells disrupt tight junctions and promote metastasis	Promoting brain metastasis and their potential as biomarkers	Lu et al. (26)
Immunomodulatory effects of exosomes in glioblastoma	Reviews the roles of exosomes in glioblastomas immunosuppressive microenvironment	Immunotherapy and cancer treatment using targeted exosomes	Benecke et al. (86)
Immunoinhibitory proteins in cancer exosomes	Role of tumor-derived exosomes in immune evasion processes	Exosome-based strategies for overcoming immunosuppression in cancers	Whiteside (87)
Diagnostic and therapeutic potential of exosomes in brain cancer	Reviews properties that make exosomes suitable for targeted drug delivery in brain tumors	Exosome-based systems to enhance therapeutic precision in brain cancer	Zhang et al. (88)
Theranostic properties of exosomes in glioblastoma	Efficacy of exosome-mediated drug delivery in glioblastoma therapies	Exosomes in personalized cancer treatment	Bălașa et al. (89)
Clinical applications of exosomes in cancer	Reviews the roles of exosomes in tumor progression, specifically their therapeutic implications	Exosomes as innovative diagnostic and therapeutic tools in clinical practice	Wu et al. (90)
Targeting cancer-associated fibroblasts with exosomes	Exosomes can influence fibroblast activity and alter the tumor microenvironment	Exosomes are dual-functional: biomarkers and therapeutic agents	Xue et al. (91)
Integrins on tumor-derived exosomes	Integrin profiles that dictate the organotrophic behavior of exosomes	Potential targeting strategies for exosome-mediated therapy	Hoshino et al. (61)
Transport of exosomes across the blood–brain barrier	Exosome interactions with the BBB and their implications in CNS diseases, including cancer	Exosomes as delivery vehicles for therapeutics	Banks et al. (92)
Recent advances in exosome applications in brain tumor treatment	Reviews the protective role of exosomes in delivering therapeutic content	Improve therapeutic stability and efficacy	Rodrigues et al. (29)
Epithelial-mesenchymal transition and exosomes	Exosome contents contribute to EMT and facilitate cancer progression	Exosomes serve as therapeutic targets in cancer therapy	Dhar et al. (93)

Exosomes can be a better nanovehicle for applications such as drug delivery, prognostic and diagnostic platforms, disease progression, and biomarker or disease monitoring platforms. The table summarizes the key focus of the study, the key findings of the reported investigation, and the implications of exosomes in theranostic modalities.

## 6 Glioblastoma derived exosomes in liquid biopsies and their clinical relevance

In addition to their therapeutic capabilities, exosomes serve as important biomarkers for brain cancer diagnosis. The cargo of exosomes derived from glioblastoma cells, including various nucleic acids and proteins, can reflect the genetic and phenotypic characteristics of the tumor, making them valuable for monitoring disease progression and treatment efficacy (48, 49). In a previous study, RNA content of exosomes, such as miRNAs and mutant p53 correlates with tumor dynamics and patient prognosis, thus assisting in clinical decision-making (50). Additionally, exosomes derived from glioblastoma can influence the tumor microenvironment by modulating immune responses and promoting tumor progression, which indicates their multifaceted roles in glioblastoma pathology (51, 52).

However, due to the intrinsic heterogeneity and small size, exosomes are restricted in their use as liquid biopsies, providing impurities and requiring less efficient separation techniques use as liquid biopsies, providing impurities and requiring less efficient separation techniques (53). Exosomes and the biomarkers they possess, including Deoxyribonucleic acid (DNA), RNA, and proteins, are commonly found in trace amounts in bodily fluids like blood and CSF, challenging to find and may provide false-negative findings (54). It can be difficult to distinguish exosomes originating from tumors from those originating from non-cancerous cells or healthy tissue. Other brain cancers or neurological disorders may also display certain biomarkers, which may lead to unrelated results. Moreover, sensitive detection techniques (such as ddPCR and sophisticated OMICS) are essential for precise quantification of biomarkers from exosomes, which increases the cost of the liquid biopsies (48).

## 7 Biosensing application of exosomes in diagnostic modalities for glioblastoma

The biomarker potential of exosomes in brain cancer is significant due to their ability to reflect the molecular characteristics of their parent cells. The cargo of exosomes, including proteins and non-coding RNAs, has been studied as a non-invasive diagnostic tool for tracking brain cancer progression and response to therapy (55). A specific exosome-derived miRNA has been shown to correlate with glioma progression, serving as a potential biomarker for disease monitoring (56). The high specificity of biomolecules in the exosome allows the differentiation between primary and metastatic brain tumors, marking their utility in guiding treatment strategies (24). Moreover, the high expression of proteins like CEMIP in exosomes has been linked to cancer prognosis, indicating the diagnostic and prognostic value these vesicles hold (29). The identification and validation of these exosome markers could lead to minimally invasive diagnostic protocols that facilitate timely interventions in brain cancer management.

## 8 RNAi with exosome as a delivery cargo

Initially, exosomes isolated from human and mouse mast cells contain inherent mRNA and microRNA, which are transported to neighboring cells and function as transportable elements (57). Therapeutically, exosomes demonstrate the capability to deliver specific molecular payloads, such as small interfering RNA (siRNA) and chemotherapeutic agents, directly to target cells. This enhances the therapeutic index and addresses drug resistance observed in many cancer treatments (58).

Exosomes are also employed for the loading of therapeutic cargo, including siRNA and chemotherapeutic agents, and have shown promising results in preclinical models. The synthetic protein nanoparticles designed to mimic exosomes significantly enhanced drug delivery to glioblastoma, illustrating a novel strategy to leverage exosome functionality for therapeutic benefit (59).

## 9 Exosome in bioinformatics: interpreting complex data

Machine learning and bioinformatics are essential for analyzing the large and intricate data from exosome profiling (60). As a multi-omics molecular reservoir, exosomes serve as a perfect biomolecule for the current library of artificial intelligence (AI) algorithms utilized in pathogenesis and treatment (61). Most studies that employ AI in EVs now focus on creating diagnostic tests; however, in the future, this data may be used to guide logical medication delivery strategies. Important changes in several genes that directly affect EV trafficking, endosomal recycling, and caveolar formation were discovered using phosphoproteomics (62). AI might also help physicians and researchers understand pathophysiology to guide medication administration. AI approaches enable advancement in the research on exosome plasmonic sensing and associated support for diagnostic applications, particularly machine learning algorithms (63). These algorithms are exceptional in flexibility and scalability for managing large datasets, making them crucial for deciphering complicated biosensing data. These AI methods are incorporated into exosome diagnostics and offer crucial methods for choosing and utilizing machine learning algorithms.

Machine learning methods are perfect for evaluating exosome profiles, as they can find trends in large datasets (64). A major advancement in early cancer detection techniques has been made through a machine learning approach for non-invasive cancer diagnosis using exosome protein markers, which achieves high accuracy in identifying cancer types with an advanced biomarker signature and sophisticated data models (65). These algorithms can categorize cancer subtypes, distinguish between carcinogenic and non-cancerous samples, and forecast the results of treatment. Furthermore, bioinformatics is a crucial tool to manage and analyze the vast amounts of data from exosome analysis and extract valuable insights from the intricate molecular profiles of exosomes (66). A paradigm shift in cancer diagnoses has been brought by the combination of liquid biopsies, nanotechnology, machine learning, and bioinformatics. This convergence makes it easier to use exosome analysis for dynamic disease monitoring and non-invasive, accurate early cancer diagnosis. Exosome-based cancer detection has the potential to become a key component of early diagnosis and personalized cancer treatment as these technologies advance, greatly influencing oncology by enhancing patient outcomes and deepening our knowledge of cancer biology.

## 10 Challenges of exosome therapeutics in brain cancer

Although exosomes possess excellent physico-chemical characteristics as a potential candidate for theranostic cargo in brain cancer diseases, they also come along with some limitations and challenges. This includes scalability, reproducibility, regulatory hurdles, and the heterogeneity of exosomes limits their clinical translation. The lack of reliable separation of exosomes with high yield and purity is one of the biggest challenges in the translatability of exosomes into the clinics. Traditional methods, such as size-exclusion chromatography, density gradient centrifugation, and ultra-centrifugation, are time-consuming, labor-intensive, and frequently co-isolate additional protein or vesicular impurities. These discrepancies hinder scalability and repeatability, which are critical for clinical translation (67). Utilizing exosomes for precise drug delivery applications is extremely difficult due to their complex nature, characterized by their varied molecular components (68). Due to its intrinsic complexity, achieving reliable and efficient drug loading into exosomes is difficult (69, 70). Additionally, exosomes naturally tend to target specific cell types or tissues, and engineered exosomes are challenging to precisely target disease locations (71). Surface-modified exosomes must be identified and bind to the targeted cells while reducing off-target effects to achieve a high degree of accuracy (72). Maintaining this fine balance between selectivity and preventing unforeseen effects is the major obstacle in the preparation of exosome-based drug delivery systems (73). The limited payload capacity of exosomes is another problematic concern for drug delivery systems. Moreover, a scalable manufacturing procedure for exosome synthesis remains a top priority. Production costs may increase significantly due to the difficulty of isolating and purifying exosomes and the requirement for modification for certain therapeutic applications (67). The cost and accessibility of exosome-based therapeutics may be impacted by the expensive nature of exosome manufacturing. The development of more economical and efficient production techniques is required for exosome therapeutics to translate its true potential into the healthcare system.

## 11 Future direction of exosome in brain cancer

Exosomes are lipid-encased, subcellular (60–80 nm) structures released into the peripheral blood from healthy and diseased cells. Exosome content varies in the physiological and pathological conditions of the same cell type or organ (74). Using exosomes as a diagnostic tool for glioma is considered a less explored area and has high potential with respect to its multitude of accolades. Commonly used biomarkers such as clinical disease activity indices, C-reactive protein (CRP), and fecal calprotectin achieve suboptimal sensitivity and specificity for brain cancer diagnosis. However, an elevated level of CRP indicates poor progression-free and overall survival in PD-1 therapy (75). The imperative need is to identify subtle disease manifestations through precise diagnostic methodologies, which are crucial for assessing disease progression or remission.

Increased sphingomyelin and saturated lipids have also been observed in the lipid profiling of glioblastoma-derived exosomes. Moreover, glioblastoma tissues have been found to contain elevated levels of sphingosine-1-phosphate (S1P), indicating that sphingolipids contribute to tumor invasion, angiogenesis, and metastasis of glioblastoma (76). Extracellular vesicles derived from glioblastoma cells with the IDH1 mutation have been reported to have a difference in lipid profile compared to normal cells (77). Building on this insight, exosomes isolated from peripheral blood referred to as peripheral blood exosomes (PBE), holds a significant potential in diagnosis and improve clinical outcomes due to their distinct lipid compositions. They offer a highly sensitive and non-invasive biomarker platform for accurately assessing disease severity. Continuous follow-up until the achievement of deep remission should be considered the definitive endpoint of therapy, alongside accurate diagnosis of the disease. In this manner, monitoring the brain cancer patients with lipid profiling of PBE, with the intent to suppress clinical cancer progression and significant remission upon therapy, is considered an important aspect in disease management. Moreover, an iRGD peptide-functionalized exosome can enhance its targeting ability by enabling its binding to integrin αvβ3, which is overexpressed in brain cancers. This engineering approach underlines the potential of exosome-based systems in coordinating targeted therapy and overcoming traditional hurdles in cancer treatment.

## 12 Conclusion

Exosomes, nanoscale extracellular vesicles secreted by cells, serve as carriers for various biomolecules, including proteins, lipids, and nucleic acids, making them valuable for targeted therapy and diagnostics in cancer management. Exosomes are emerging as key players in the progression of brain cancer, influencing both therapeutic approaches and diagnostic strategies. Their role in facilitating metastasis and altering the tumor microenvironment presents both challenges and opportunities for intervention. Advances in exosome engineering provide exciting avenues for enhancing targeted drug delivery systems, potentially transforming therapeutic outcomes (50). Meanwhile, as biomarkers, exosomes offer new hope for improved disease monitoring and personalized treatment strategies (55). Continued research into the multifaceted roles of exosomes will be crucial in shaping future brain cancer therapies. The evaluation of exosome-integrin interactions elucidates the mechanisms through which cancer cells exploit exosomes to their advantage. Integrin expression patterns on exosomes can serve as biomarkers for predicting organotrophic metastasis, underscoring the importance of targeting these pathways in brain-related malignancies (26, 43).

The therapeutic potential of exosomes is expanding with advances in engineering techniques, particularly in enhancing their specificity and uptake in brain tumors. The use of peptides like the iRGD enhances the targeting ability of exosomes by facilitating their binding to integrin αvβ3, which is overexpressed in various tumors (40). The integration of iRGD peptide technology with exosome-based platforms presents a groundbreaking opportunity in brain cancer research and treatment. This multifaceted approach may lead to better-targeted therapies that improve patient outcomes while minimizing systemic toxicity associated with conventional treatments.

The theranostic potential of exosomes in brain cancer is vast, bridging the gap between diagnostic applications and therapeutic strategies. Exosomes not only provide valuable biomarkers for monitoring disease progression but can also be engineered to enhance drug delivery and efficacy in targeting brain tumors. As ongoing research continues to unveil the complexities of exosome biology, their integration into clinical practice could significantly improve outcomes for patients with brain cancer. Future investigations focused on optimizing surface engineering of exosomes, understanding their interactions within the tumor microenvironment, and validating their clinical utility will be integral in translating these findings into meaningful therapies.
