# Supplementary material for: Therapeutic and diagnostic implications of exosomes as natural nanoparticles: a new paradigm in brain cancer disease management
Source: Front Med (Lausanne). 2025 Jul 21;12:1599392. doi: 10.3389/fmed.2025.1599392 (PMC12319004; doi:10.3389/fmed.2025.1599392)
Supplement: Supplementary file 1 [file Table_1.docx]

Supplementary Table 1. Summary of Search Strategies and Retrieved Records

| Target Database | Summary of Search Strategy | Retrieved Articles |
| --- | --- | --- |
| Scopus | TITLE-ABS-KEY ( glioblastoma AND exosomes AND brain AND cancer ) AND PUBYEAR > 2008 AND PUBYEAR < 2025 AND ( LIMIT-TO ( PUBSTAGE , "final" ) ) AND ( LIMIT-TO ( DOCTYPE , "ar" ) OR LIMIT-TO ( DOCTYPE , "re" ) ) AND ( LIMIT-TO ( LANGUAGE , "English" ) ) AND ( LIMIT-TO ( EXACTKEYWORD , "Glioblastoma" ) OR LIMIT-TO ( EXACTKEYWORD , "Exosome" ) OR LIMIT-TO ( EXACTKEYWORD , "Exosomes" ) OR LIMIT-TO ( EXACTKEYWORD , "Brain Neoplasms" ) OR LIMIT-TO ( EXACTKEYWORD , "Brain Tumor" ) OR LIMIT-TO ( EXACTKEYWORD , "Glioma" ) ) (LIMIT-TO (LANGUAGE, “English”)) | 236 |
| PubMed | (("glioblastoma"[MeSH Terms] OR "glioblastoma"[All Fields] OR "glioblastomas"[All Fields]) AND ("exosomal"[All Fields] OR "exosomes"[MeSH Terms] OR "exosomes"[All Fields] OR "exosome"[All Fields] OR "exosomic"[All Fields]) AND ("brain neoplasms"[MeSH Terms] OR ("brain"[All Fields] AND "neoplasms"[All Fields]) OR "brain neoplasms"[All Fields] OR ("brain"[All Fields] AND "cancer"[All Fields]) OR "brain cancer"[All Fields])) AND ((classicalarticle[Filter] OR preprint[Filter] OR review[Filter]) AND (humans[Filter] OR animal[Filter]) AND (english[Filter])) Translations glioblastoma: "glioblastoma"[MeSH Terms] OR "glioblastoma"[All Fields] OR "glioblastomas"[All Fields] OR "glioblastoma's"[All Fields] exosomes: "exosomal"[All Fields] OR "exosomes"[MeSH Terms] OR "exosomes"[All Fields] OR "exosome"[All Fields] OR "exosomic"[All Fields] brain cancer: "brain neoplasms"[MeSH Terms] OR ("brain"[All Fields] AND "neoplasms"[All Fields]) OR "brain neoplasms"[All Fields] OR ("brain"[All Fields] AND "cancer"[All Fields]) OR "brain cancer"[All Fields] | 61 |
| Web of Science: (Core Collection) | ((ALL=(exosomes)) AND ALL=(brain cancer)) AND ALL=(glioblastoma) and Article or Review Article (Document Types) and English (Languages) and 2024 or 2023 or 2022 or 2021 or 2020 or 2019 or 2018 or 2017 or 2016 or 2015 or 2014 or 2013 or 2012 or 2011 (Publication Years) | 302 |
